# Differential Cytoprotective Effect of Resveratrol and Its Derivatives: Focus on Antioxidant and Autophagy-Inducing Effects

**DOI:** 10.3390/ijms252011274

**Published:** 2024-10-20

**Authors:** Kamilla Varga, Alexandra Paszternák, Virág Kovács, Annamária Guczogi, Noémi Sikur, Dimitrisz Patakfalvi, Fruzsina Bagaméry, Éva Szökő, Tamás Tábi

**Affiliations:** 1 Department of Pharmacodynamics, Semmelweis University, 4 Nagyvárad tér, H-1089 Budapest, Hungary; varga.kamilla@phd.semmelweis.hu (K.V.); paszternak.alexandra@semmelweis.hu (A.P.); sikur.noemi@stud.semmelweis.hu (N.S.); bagamery.fruzsina@semmelweis.hu (F.B.); szoko.eva@semmelweis.hu (É.S.); 2 Center for Pharmacology and Drug Research & Development, Semmelweis University, 26 Üllői út, H-1085 Budapest, Hungary

**Keywords:** resveratrol, cytoprotection, structure–activity relationship, autophagy, mitochondria, antioxidant

## Abstract

Numerous beneficial effects of resveratrol were reported; however, its pharmacological profile is contradictious. Previously, we have demonstrated that resveratrol has a dose-dependent cytoprotective effect and the essential role of autophagy induction was demonstrated. Resveratrol suffers from unfavorable pharmacokinetics, hindering its clinical use. Our aim was to study the cytoprotective effect of resveratrol derivatives to better understand structure–activity relationships that may facilitate the development of compounds with better druglike characteristics. Serum-deprivation-induced caspase activation, free radical generation, mitochondrial membrane depolarization and autophagy were detected in the presence of resveratrol analogs with different oxidation states on mouse embryonal fibroblasts. Distinct cytoprotective mechanisms of the examined compounds were revealed. Monomethyl resveratrol had similar potency to resveratrol (EC_50_: 85.3 vs. 84.2 μM); however, autophagy induction was not essential for its cytoprotective effect. Oxyresveratrol was found to be a strong antioxidant that can induce direct cytoprotection rather than autophagy. Trimethyl-resveratrol, lacking free hydroxyl groups, induced damage that was too significant and hardly compensated by the activation of cytoprotective machineries, and caspase activation was reduced by only 24.5%. Based on our results, methylation of resveratrol reduces its antioxidant activity, while autophagy induction can still contribute to its cytoprotective effect. The introduction of an additional hydroxyl group, however, augments the antioxidant properties, inducing cytoprotection without autophagy induction.

## 1. Introduction

Resveratrol, a natural compound found in red wine [1], has received widespread attention for decades because of its beneficial pharmacological effects like protection against age-related disorders, such as neurodegenerative [2,3], metabolic [4] and cardiovascular diseases [5,6]. It has been shown to be capable of protecting cells against various damages, e.g., oxidative stress [7] and hypoxia [8]. It could improve cell viability [9] and prevent cell death by modulating apoptotic processes [10]. On the other hand, the compound was also reported to have proapoptotic and anticancer activities [11,12]. Studies suggest its effect is significantly influenced by the investigated cell type; its cytotoxic properties have mainly been reported on cancer cell lines, while its cytoprotective effect is rather shown on non-transformed cell cultures [13,14]. As we intended to study the cytoprotective effect of resveratrol, we chose a primary cell culture, mouse embryonal fibroblast (MEF), as our model system.

Several mechanisms behind the beneficial effects of resveratrol have been demonstrated. It is a polyphenolic stilbene derivative, a chemical structure that has been reported to possess antioxidant [15,16] and pro-oxidant [14,17] properties alike. The elimination of reactive oxygen species (ROS) by resveratrol may derive from a combination of its direct scavenger activity [18] and modulation of expression of endogenous antioxidant enzyme systems [19]. Resveratrol-induced ROS generation, on the other hand, has been suggested to be the consequence of compromised mitochondrial respiration [20,21]; however, interaction with metal ions resulting in an increased free radical formation has been reported as well [22].

The underlying mechanism of the effect of resveratrol is still being debated since it has been shown to be able to modulate various signaling pathways such as MAP kinases (ERK1/2 [23] and p38 [24]), PDE [25], PI3K [26], and PPARγ [27], and influence intracellular receptors and transcription factors including estrogen receptors [28] or aryl hydrocarbon receptors [29], FOXO [30], Nrf2 [26] and NF-κB [31]. However, their most important targets in primary cells could be SIRT1 and AMPK, which are responsible for the beneficial effects of caloric restriction [32]. Several lines of evidence support that the cytoprotective effect of resveratrol can be prevented by inhibiting either SIRT1 or AMPK activity [33]; however, because they can activate each other, it is difficult to determine if they are the primary target [32]. Complex interaction with the SIRT1-AMPK pathway could explain the potentiation of mitochondrial biogenesis via PGC-1α [34] and the induction of autophagy by inhibiting mTOR [35]. The exact point at which resveratrol initiates autophagy and whether it induces bulk or selective autophagy are also debated [2]. Furthermore, the potential mechanism of resveratrol could be the result of the mitochondrial respiratory chain uncoupling effect that depletes both NADH and ATP, leading to the activation of these two key metabolic regulator proteins [21].

In our previous works, resveratrol was shown to have a strong, concentration-dependent inhibitory effect on serum-deprivation-induced caspase 3 activation in a primary cell culture [24,36]. We have also demonstrated that its effect mimics preconditioning since cytoprotection was mediated by the induction of mild stress. According to our hypothesis, resveratrol can induce weak mitochondrial damage that is counterbalanced by its antioxidant activity, resulting in the activation of protective machineries, primarily autophagy, which was found to be essential for the prevention of caspase activation [36].

Although resveratrol possesses a wide range of potential pharmacological effects, its application is considerably hindered by its unfavorable pharmacokinetic profile. It suffers from poor bioavailability mainly due to its low metabolic stability [37]. Methylation of phenolic hydroxyl groups of resveratrol was reported to result in improved bioavailability [38]. Moreover, improved chemical stability of the methylated derivatives was also reported [39], which can be a significant advantage during formulation and storage of dosage forms. Mono-, di- and trimethyl derivatives of resveratrol all occur naturally and are reported to have some similar pharmacological activities to their parent compound [40,41]. On the other hand, the phenolic hydroxyl groups contribute significantly to the pharmacological effects of stilbenes as well. It was reported that compounds with a different number and position of hydroxyl groups have different antioxidant activity [42]. Among its hydroxyl groups, the 4′ one plays the most important role in the elimination of free radicals; therefore, its presence significantly contributes to the antioxidant activity of resveratrol [42]. Furthermore, the introduction of additional phenolic hydroxyl groups has been shown to increase its antioxidant efficacy [43,44]. Oxyresveratrol is another naturally occurring compound that has an additional hydroxyl group at position 2′. It is reported to be a strong antioxidant [45] and have neuro- and cytoprotective effects [46] similarly to resveratrol.

Although some investigations aiming for better clarification of the structure–activity relationship of resveratrol and related stilbenes have been conducted to improve its drug-like characteristics, the majority of the studies were performed on transformed cancer cell lines and only very few studies dealt with the cytoprotective mechanisms in non-transformed cells.

**Scheme 1:**
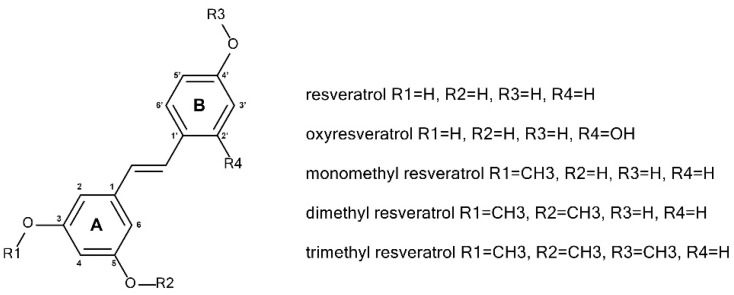
Chemical structure of the examined compounds. Resveratrol: 3,4′,5-trihydroxy-*trans*-stilbene, oxyresveratrol: 2′,3,4′,5-tetrahydroxy-*trans*-stilbene, monomethyl resveratrol: 3-methoxy-4′,5-dihydroxy-*trans*-stilbene, trimethyl resveratrol: 3,4′,5-trimethoxy-*trans*-stilbene, dimethyl resveratrol: 3,5,-dimethoxy-4′-hydroxy-*trans*-stilbene.

Our aim, thus, was to directly compare the cytoprotective effect of resveratrol and its analogs with different oxidation states in the previously used model to better understand the contribution of hydroxyl groups to their caspase-3-activation-preventing effect and their mechanism of action. The chemical structures of the examined compounds are shown in Scheme 1.

## 2. Results

### 2.1. Effect of Resveratrol Derivatives on LDH Release

**Figure 1:**
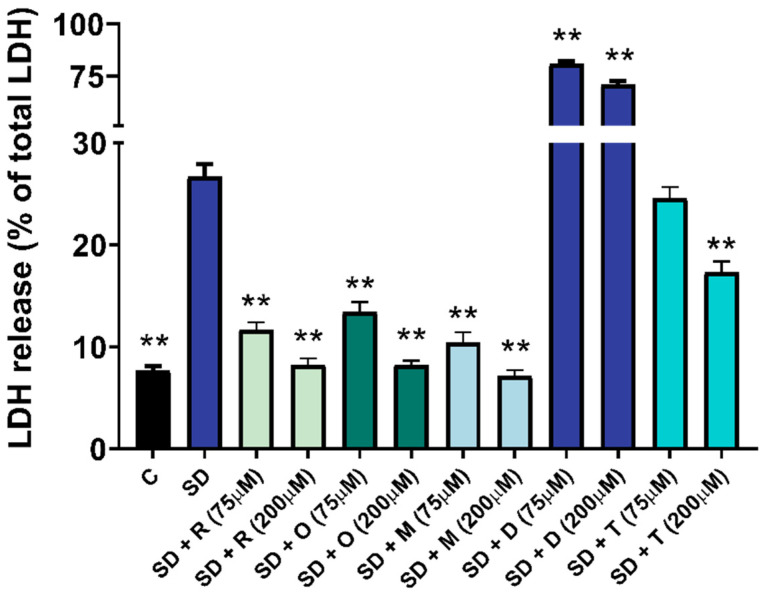
Effect of resveratrol and its analogs on LDH release induced by 6 h serum deprivation in MEF. Serum-deprivation-induced LDH release was significantly reduced by resveratrol, monomethyl resveratrol, oxyresveratrol and trimethyl resveratrol, while it was strongly augmented by dimethyl resveratrol. C: control, SD: serum deprivation, R: resveratrol, O: oxyresveratrol, M: monomethyl resveratrol, D: dimethyl resveratrol, T: trimethyl resveratrol n = 3, **: *p* < 0.001 vs. SD.

The effect of resveratrol analogs on serum-deprivation-induced cell death was studied by assaying the loss of membrane integrity by the lactate dehydrogenase (LDH) release method. Resveratrol, oxyresveratrol and monomethyl resveratrol showed a highly effective cytoprotective property as the compounds completely prevented the serum-deprivation-induced elevation in the LDH release. Cytoprotection by trimethyl resveratrol was inferior compared to that of the parent compound since only partial reduction in LDH release was observed. Surprisingly, dimethylated resveratrol was highly cytotoxic with about 90% loss of membrane integrity in response to the compound; therefore, it could not be used for further experiments (Figure 1).

### 2.2. Effect of Resveratrol Analogs on Caspase 3 Activity

**Figure 2:**
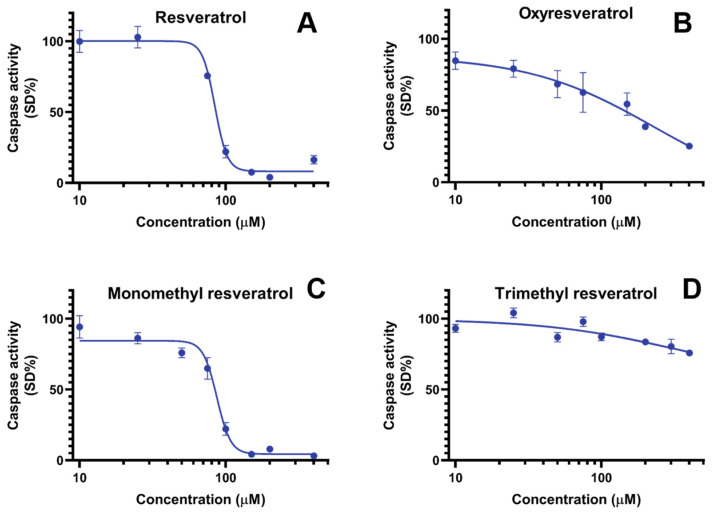
Effect of resveratrol (**A**), oxyresveratrol (**B**), monomethyl resveratrol (**C**) and trimethyl resveratrol (**D**) on caspase 3 activation induced by 3 h serum deprivation in MEF. Caspase 3 activity in the control cells was 0.0228 ± 0.000598 nmol/mg, which was increased to 0.6019 ± 0.000809 nmol/mg level in response to 3 h serum deprivation. Concentration-dependent effect of the concomitantly administered resveratrol derivatives on caspase 3 activation was characterized by using fluorogenic caspase substrate. EC_50_ values for resveratrol: 84.20 µM (95% CI: 70.39 µM–98.02 µM), monomethyl resveratrol: 85.32 µM (95% CI: 75.74 µM–98.07 µM), oxyresveratrol: 232.9 µM (95% CI: 84.02 µM–587.6 µM), trimethyl resveratrol: >400 µM; n = 4 in each measurement.

Contribution of apoptosis to serum-deprivation-induced cell death was detected by measuring caspase 3 activity in cells treated simultaneously with resveratrol and resveratrol analogs (Figure 2). Because caspase activation precedes loss of membrane integrity, it was detected after 3 h of serum deprivation. Three of the examined compounds significantly reduced caspase 3 activation on a concentration-dependent manner. Resveratrol and monomethyl resveratrol had similar efficacy and potency, their 50% inhibitory concentration (EC_50_) values did not differ significantly (84.20 µM and 85.32 µM, respectively), and both of them were capable to reduce caspase 3 activity to the control level at around 150 µM concentration (Figure 2A,C). Oxyresveratrol showed impaired efficacy and potency compared to resveratrol; its EC_50_ value was around 115 µM and it was not able to completely prevent caspase 3 activation at even the highest studied concentration of 400 µM. Furthermore, the slope of its concentration–response curve was considerably less steep (Figure 2B). Trimethylated resveratrol caused a slight, but significant caspase 3 inhibition; even at 400 µM concentration, it induced only a 24.5 ± 1.5% reduction (Figure 2D).

### 2.3. Effect of Resveratrol Derivatives on ROS Production

**Figure 3:**
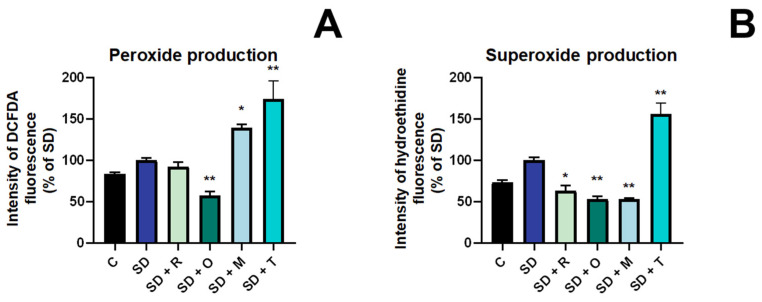
Effect of resveratrol and its analogs on ROS production secondary to 3 h serum deprivation. The tested compounds were used in 200 µM concentration. (**A**) Peroxide production measured by DCFDA staining. (**B**) Superoxide production measured by hydroethidine staining. Oxyresveratrol showed an antioxidant effect in both tests, and trimethyl resveratrol increased the free radicals’ production, while monomethyl resveratrol showed an antioxidant effect on superoxide and facilitated hydrogen peroxide generation. SD: serum deprivation, R: resveratrol, O: oxyresveratrol, M: monomethyl resveratrol, T: trimethyl resveratrol. n = 5, *: *p* < 0.05 vs. SD, **: *p* < 0.001 vs. SD.

Polyphenolic compounds are known for their antioxidant properties that may contribute to their cytoprotective activity, and thus free radical production in the presence of resveratrol and its analogs was also analyzed. Although resveratrol failed to significantly decrease peroxide production compared to the serum-deprived group, it showed a slight superoxide reducing effect. Oxyresveratrol with an additional free hydroxyl group showed direct antioxidant activity and effectively reduced both peroxide and superoxide production, while trimethylated resveratrol bearing no free hydroxyl groups exhibited considerable pro-oxidant effect. Interestingly, although monomethyl resveratrol increased the production of peroxide residues, it also showed a strong superoxide scavenging effect (Figure 3). Since peroxide residues are found in almost all organelles, while superoxide production is rather mitochondria-specific, we further investigated the effect of the compounds on the mitochondria.

### 2.4. Effect of Resveratrol Analogs on Mitochondrial Membrane Depolarization

**Figure 4:**
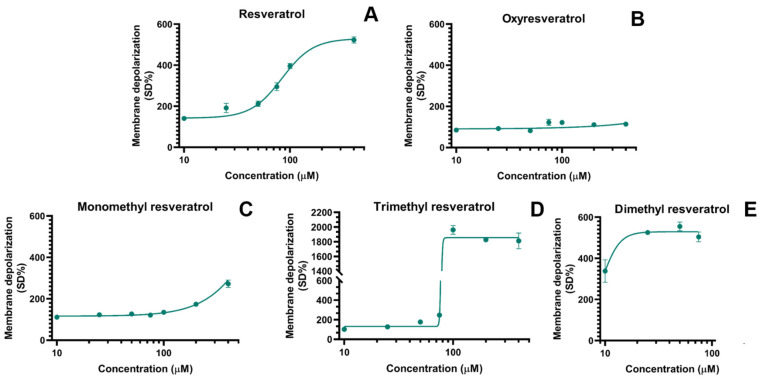
Effect of resveratrol (**A**), oxyresveratrol (**B**), monomethyl resveratrol (**C**), trimethyl resveratrol (**D**) and dimethyl resveratrol (**E**) on mitochondrial membrane depolarization induced by 3 h of serum deprivation in MEF. Serum deprivation itself induced only a mild alteration in the JC1 green-to-red fluorescence ratio (control: 0.259 ± 0.013, SD: 0.325 ± 0.018, *p* = 0.198). Resveratrol and its analogs induced concentration-dependent mitochondrial membrane depolarization. EC_50_ values for resveratrol: 83.9 µM (95% CI: 72.43 µM–95.38 µM), monomethyl resveratrol: >400 µM, oxyresveratrol: >400 µM, trimethyl resveratrol: 77.7 µM (95% CI: 68.91 µM–86.49 µM), dimethyl resveratrol: 8.73 µM (95% CI: 5.35 µM–12.11 µM), n = 5 in each measurement.

In line with our previous results [36], resveratrol induced mitochondrial depolarization with the very same potency (EC_50_ value is 83.9 µM) as its caspase-3-activity-reducing effect (Figure 4A). Monomethylated resveratrol also induced mitochondrial membrane depolarization, but only in a considerably higher concentration compared to its caspase 3 inhibitory activity; its estimated EC_50_ value was more than 400 µM (Figure 4C). Among the examined analogs, the trimethylated one caused the most significant mitochondrial depolarization, and an increase of about twenty-fold in JC-1 staining was detected (Figure 4D) compared to the fivefold elevation seen with resveratrol. The effect of oxyresveratrol on mitochondrial membrane potential was negligible (Figure 4B). Dimethyl resveratrol caused considerable mitochondrial damage even in low concentrations (EC_50_ value was 8.73 µM), which might contribute to its strong cytotoxic effect (Figure 4E).

### 2.5. Effect of Resveratrol Analogs on Mitochondrial Mass

**Figure 5:**
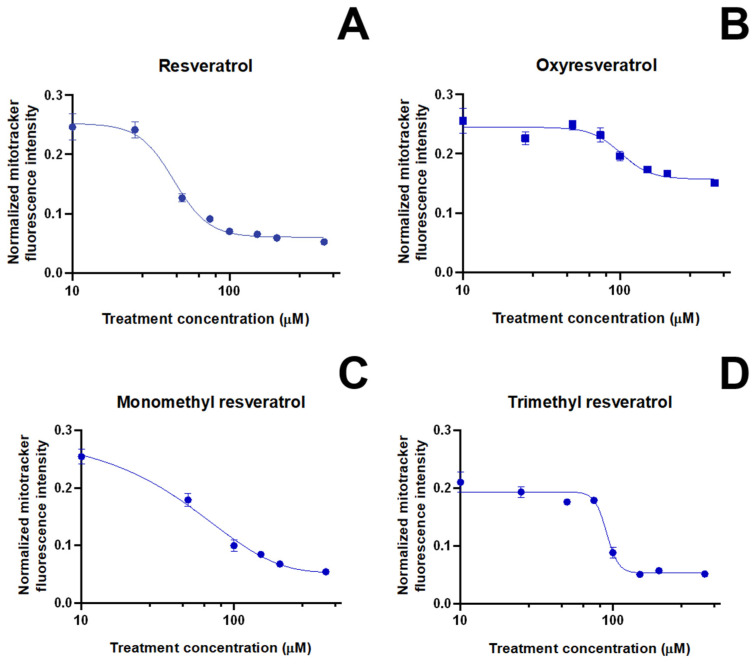
Effect of resveratrol (**A**), oxyresveratrol (**B**), monomethyl resveratrol (**C**), trimethyl resveratrol (**D**) on mitochondrial mass after 3 h serum deprivation in MEF. Mitotracker green fluorescence intensity vales were normalized by Hoechst 33342 staining. Serum deprivation decreased the number of mitochondria significantly (control: 0.385 ± 0.028, SD: 0.263 ± 0.017, n = 4, *p* = 0.0336). Resveratrol and its analogs induced concentration-dependent mitochondrial mass decrease. EC_50_ values for resveratrol: 40.04 µM (95% CI: 35.58 µM–44.50 µM), monomethyl resveratrol: 63.14 µM (95% CI: 43.29 µM–82.99 µM), oxyresveratrol: 290.7 µM (95% CI: 70.83 µM–524.62 µM), trimethyl resveratrol: 91.95 µM (95% CI: 88.32 µM–104.74 µM), n = 4 in each measurement.

To investigate whether the change in mitochondria number contributed to the observed mitochondrial membrane depolarization effect, we measured the mitochondrial mass. Contrary to the minor effect on mitochondrial membrane depolarization, serum deprivation significantly decreased the mitochondrial density. All resveratrol derivatives had a dose-dependent mitochondrial mass-depleting effect, though with different efficacy and potency. Resveratrol potently induced a reduction in mitochondrial mass (Figure 5A), as its EC_50_ value was about half of that seen in case of membrane depolarization (40.04 µM vs. 83.9 µM). In line with its effect on membrane depolarization, oxyresveratrol showed only a slight mitochondrial mass reduction (Figure 5B), suggesting the effect on the mitochondria is secondary to other cytoprotective effects. Monomethyl resveratrol was very efficient in reducing mitochondrial density (Figure 5C) in contrast to its slight effect on mitochondrial membrane potential. Although the slope of the curve was inferior, its potency was similar to that seen in case of caspase-activity-reducing effect. Trimethylated resveratrol exhibited a significant reduction in mitochondrial mass (Figure 5D); however, it was less pronounced compared to its strong impact on depolarization.

### 2.6. Effect of Resveratrol Derivatives on Accumulation of Acidic Vacuoles

**Figure 6:**
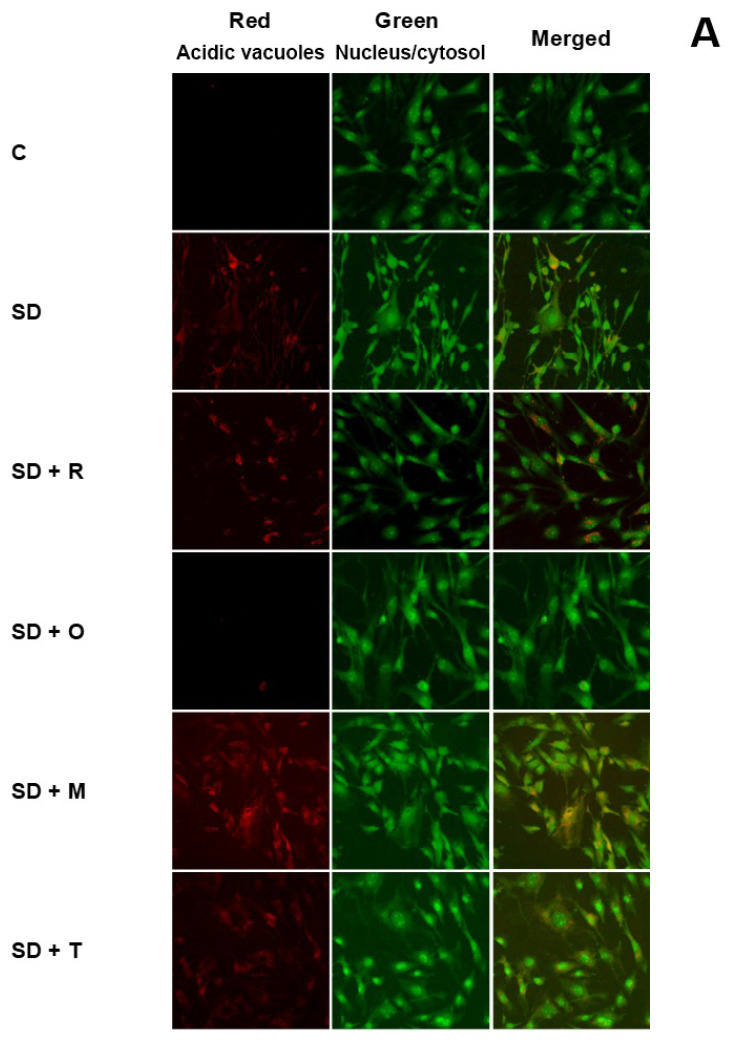

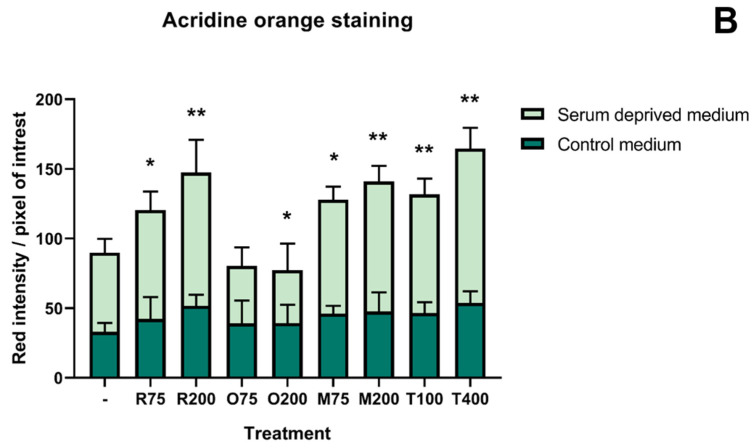
Effect of resveratrol and its analogs on accumulation of acidic vacuoles. Representative images (**A**) and relative intensity of staining (**B**) are shown. Serum-deprivation-induced accumulation of acidic vacuoles was significantly augmented by resveratrol, monomethyl resveratrol and trimethyl resveratrol in a concentration-dependent mode. SD: serum deprivation, R: resveratrol, O: oxyresveratrol, M: monomethyl resveratrol, T: trimethyl resveratrol and their concentration in µM. Acridine orange was used to visualize acidic vacuoles that are stained red, while nucleic acids in nucleus and cytosol are stained green. Photos are taken by epifluorescent phase contrast microscope with 10× objective and 10× ocular magnification. n = 4, *: *p* < 0.05 vs. SD, **: *p* < 0.001 vs. SD.

According to our previous conclusion, resveratrol facilitates autophagy that plays a central role in its cytoprotective effect [36] and may contribute to the reduction in mitochondrial mass observed in our present experiments. Autophagic processes in the presence of resveratrol analogs were thus assessed by multiple methods. Resveratrol analogs were studied in two concentration levels near the ones inducing half maximum and maximum caspase-3-activation-preventing effect. First, autophagolysosomes were visualized by acridine orange staining. Serum deprivation significantly increased the quantity of acidic vacuoles. Similarly to resveratrol, its mono- and trimethylated derivatives induced a further accumulation of autophagolysosomes in the presence of serum deprivation, suggesting the induction of autophagy. Contrary to the other examined compounds oxyresveratrol failed to increase the quantity of autophagolysosomes, and in high concentrations, it even caused a slight but significant reduction in the intensity of acridine orange staining. No significant alteration was detected in serum-supplemented cultures (Figure 6).

**Figure 7:**
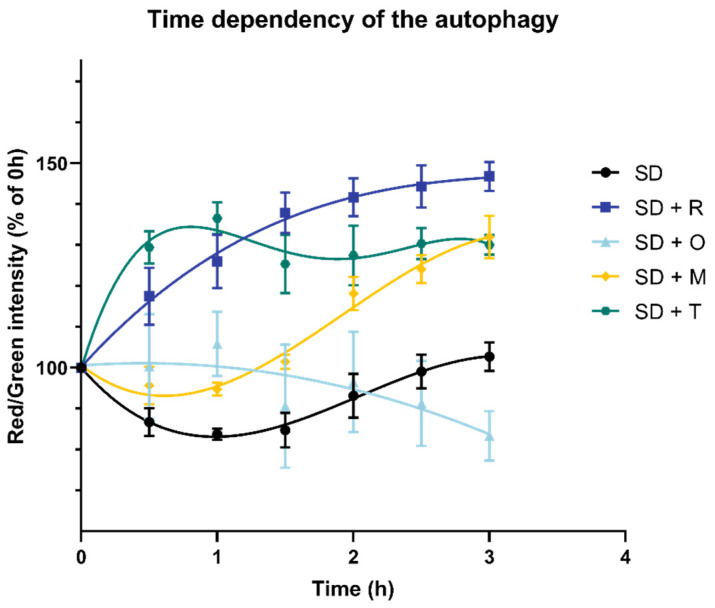
Effect of resveratrol and its analogs on the time course of accumulation of acidic vacuoles. The tested compounds were used in 200 µM concentration. The effect of resveratrol and trimethyl resveratrol was developed rapidly, while that of monomethyl resveratrol was delayed by about 1.5 h. SD: serum deprivation, R: resveratrol, O: oxyresveratrol, M: monomethyl resveratrol, T: trimethyl resveratrol, n = 4 in each measurement.

Studying the time course of accumulation of acidic vacuoles by on-line red-to-green ratio analysis [47] indicated that resveratrol and its trimethylated derivative caused a rapid increase in acridine orange staining, while the effect of monomethyl resveratrol was delayed by 1.5–2 h, indicating different mechanisms of autophagy induction. Oxyresveratrol caused a slight decrease in staining only after 3 h of serum deprivation (Figure 7).

### 2.7. Effect of Resveratrol Analogs on Specific Autophagy Marker Proteins

**Figure 8:**
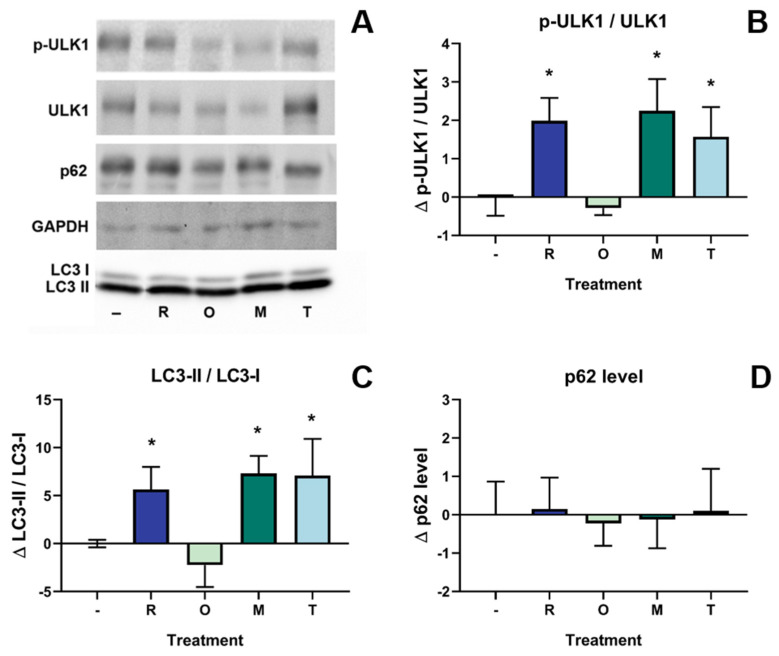
Effect of resveratrol and its analogs on the phosphorylation of ULK1 (**B**), lipidation of LC3 (**C**) and degradation of p62 (**D**) in serum-deprived cells. Panel (**A**) shows a representative Western blot, the (**B**–**D**) panels show the difference of treated and untreated cells. The tested compounds were used in 200 µM concentration. Resveratrol, monomethyl resveratrol and trimethyl resveratrol increased ULK1 phosphorylation and LC3 lipidation, while oxyresveratrol had no effect. None of the tested compounds affected the degradation of p62. R: resveratrol, O: oxyresveratrol, M: monomethyl resveratrol, T: trimethyl resveratrol. n = 6, *: *p* < 0.05 vs. SD.

To better understand the mechanism of autophagy induction by resveratrol derivatives, the levels of three autophagy-related proteins were determined by Western blot. Phosphorylation of ULK1 at Ser555 is an indicator of autophagy initiation phase [48]. Resveratrol, monomethyl and trimethyl resveratrol increased ULK1 phosphorylation, which indicates the induction of autophagy at or before the initiation phase (Figure 8B).

The ratio of LC3-II/LC3-I that is proportional to the extent of the elongation phase [48] was elevated by all derivatives with the exception of oxyresveratrol, which is consistent with increased autophagy in response to resveratrol derivatives but oxyresveratrol (Figure 8C). These results also suggest that oxyresveratrol does not augment autophagy even at its later stage.

The degradation of p62/SQSTM1 is used as a marker of selective autophagy, such as mitophagy, because it selectively recruits ubiquitinated proteins and organelles and degrades along with them in the autophagolysosomes [49]. No alteration was detected in p62 levels, indicating that selective autophagy is probably not involved in the cytoprotective effect of resveratrol derivatives (Figure 8D).

### 2.8. Effect of Autophagy Inhibitors on the Protective Effect of Resveratrol and Its Analogs

**Figure 9:**
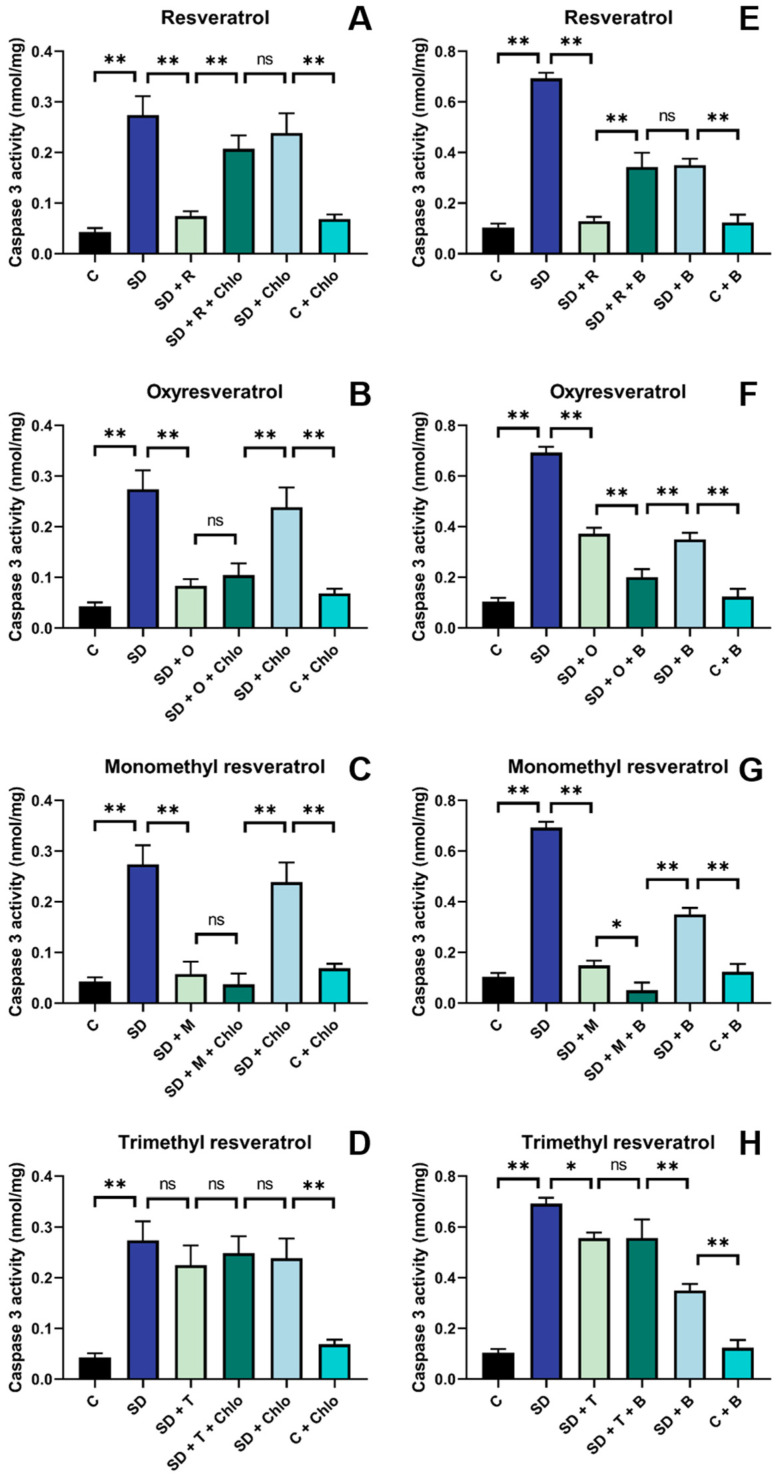
Effect of chloroquine (**A**–**D**) and bafilomycin (**E**–**H**) on the serum-deprivation-induced caspase-3-activation-preventing activity of resveratrol (**A**,**E**), oxyresveratrol (**B**,**F**), monomethyl resveratrol (**C**,**G**), trimethyl resveratrol (**D**,**H**). The tested compounds were used in 200 µM concentration. Autophagy inhibitors significantly reduced the caspase-3-activation-preventing effect of resveratrol only. SD: serum deprivation, R: resveratrol, O: oxyresveratrol, M: monomethyl resveratrol, T: trimethyl resveratrol, Chlo: chloroquine, B: bafilomycin. n = 4, *: *p* < 0.05, **: *p* < 0.001, ns: not significant.

In order to confirm the contribution of autophagy induction to the cytoprotective effect of resveratrol and its analogs, two well-established autophagy inhibitors, chloroquine and bafilomycin, were used simultaneously with the tested compounds and serum-deprivation-induced caspase 3 activation was determined. In line with our previous results [36], chloroquine treatment prevented the protective effect of resveratrol (Figure 9A). Moreover, the other autophagy inhibitor, bafilomycin, also significantly reduced its effect on caspase 3 activation (Figure 9E). These results further confirm the involvement of autophagy induction in the cytoprotective effect of resveratrol.

Although, monomethyl resveratrol had similar caspase-3-activation-preventing and autophagy-inducing effects to those of resveratrol, its protective effect was not reversed by either autophagy inhibitors (Figure 9C,G). These data indicate that though monomethyl resveratrol induces autophagy, this process does not significantly contribute to its caspase-activation-preventing effect.

In case of oxyresveratrol and trimethyl resveratrol, the autophagy inhibitors did not significantly reduce their effect on caspase 3 activation, and bafilomycin slightly improved the cytoprotective effect of oxyresveratrol (Figure 9B,F).

## 3. Discussion and Conclusions

Previously, we have shown that resveratrol possesses a preconditioning-like cytoprotective effect against serum-deprivation-induced damage. This may have a significant potential for the development of neuro- and cardioprotective medications, a field of drug research with high demand for effective treatments [50]. Resveratrol, similarly to several other herbal remedies, is characterized by poor pharmacokinetics; thus, it is inappropriate for further drug development [37]. Its derivatives with improved pharmacological and pharmacokinetic properties, however, can serve as lead molecules for future medication research.

In the present study, we showed that oxyresveratrol and monomethylated resveratrol can significantly prevent the serum-deprivation-induced caspase activation in MEF. Monomethyl resveratrol has similar potency and efficacy to resveratrol, while oxyresveratrol was found to be inferior in both parameters. Moreover, investigation of the mechanism of cytoprotective effect of the three compounds revealed more fundamental differences.

Contrary to resveratrol, oxyresveratrol may reduce the serum-deprivation-induced noxious processes directly probably by scavenging free radicals rather than removing damaged mitochondria by autophagy induction. Its elongated concentration–response curve also suggests a less precisely regulated or non-receptorial mechanism, such as antioxidant effect. It was previously shown that the introduction of an additional phenolic hydroxyl group to the resveratrol molecular core augmented the antioxidant activity [43,44]. Furthermore, oxyresveratrol was also reported to be a strong antioxidant [51], capable of reversing hydrogen peroxide-induced apoptosis in primary HLECs endothelial cells [52] and tert-butyl hydroperoxide led to cell death of HepG2 hepatocytes [53]. In the latter study, the prevention of mitochondrial depolarization was also shown [53], which is in accordance with our present results.

Indeed, oxyresveratrol was the only examined compound that failed to induce autophagy in the present study. Although it was previously reported to be capable of activating autophagy in various cell types [54,55,56], in these works, the effect of oxyresveratrol was investigated in the absence of cell damage. In the present experiments, serum deprivation itself augmented the autophagic process significantly and oxyresveratrol was ineffective in further facilitating it, contrary to the other examined resveratrol derivatives.

Monomethyl resveratrol shared more similarities with resveratrol. They have comparable effects on caspase activation prevention and mitochondrial mass reduction, though their mechanisms were found to be distinct. Compared to resveratrol, its monomethyl derivative caused less mitochondrial damage but induced more pronounced peroxide generation combined with a reduction in superoxide production. In line with these results, its inferior free radical scavenger activity compared to resveratrol was previously established in cell-free systems [57,58] and it was found to have a pro-oxidant effect in THP-1 macrophages by itself and to be able to further increase the ROS production induced by pyocyanin [59]. On the other hand, the antioxidant activity of monomethyl resveratrol has been established as well [60], which correlates with our data on superoxide production. The discrepancy between these two types of residues could be explained by a mitochondria-specific antioxidant mechanism that could also explain our results on mitochondrial mass reduction with little to no effect on mitochondrial membrane depolarization.

Although monomethyl resveratrol effectively induced autophagy, according to our results, this effect did not play a role in the prevention of caspase activation contrary to resveratrol. Analyzing the time course of generation of acidic vacuoles indicated a considerably delayed activation of autophagy that may explain the lack of its contribution to the cytoprotective effect of monomethyl resveratrol. Recently, Allen et al. reported that monomethyl resveratrol and resveratrol both protected SH-SY5Y cells against dopamine neurotoxicity by enhancing ERK signaling; however, the onset of the latter effect of monomethyl resveratrol was slower [60]. The delayed onset of some effects of monomethyl resveratrol may raise the possibility of its conversion to resveratrol, which might also contribute to its actions. However, the autophagy-induction-independent caspase-3-activation-preventing effect of monomethyl resveratrol suggests an action distinct from that of resveratrol. Although its selective mitochondrial superoxide scavenger activity may play a central role, a better understanding of its cytoprotective mechanism requires further studies.

Trimethylated resveratrol showed very limited cytoprotective effect against serum-deprivation-induced caspase 3 activation that was accompanied by highly significant mitochondrial depolarization and ROS generation. The lack of its antioxidant activity can be explained by the absence of free hydroxyl groups that are claimed to play an important role in the scavenger activity of stilbenoids [42]. The paramount importance of 4′ hydroxyl group of resveratrol in its antioxidant activity was reported in [42], which is in line with the present results of different ROS production observed in the case of monomethyl and trimethyl resveratrol. The diminished scavenger activity may also contribute to its strongly aggravated mitochondrium-damaging effect that is not accompanied by a similarly strong mitochondrial mass reduction. We can speculate that it may interfere with mitochondrial respiration but, due to the lack of antioxidant activity, the more considerable free radical generation may further impair mitochondrial function entering a vicious cycle. The rapid induction of autophagy may prevent excessive cell damage, although its involvement in the weak cytoprotective effect of trimethyl resveratrol requires further investigation.

Interestingly, dimethylated resveratrol was found to induce a strong cytotoxic effect contrary to all other examined compounds. Although most of the studies reported similar actions to those of resveratrol [40], Peng et al. recently showed its proapoptotic effect in an induced pulmonary fibrosis model but not on healthy pulmonary epithelial cells, suggesting its tissue-specific action, i.e., selective damage to fibroblasts or fibroblast-like cells [61].

The major limitation of the present work is its in vitro design. Although the experiments were planned carefully and we used non-transformed cells to minimize the bias deriving from cell culture studies, the observed effects of resveratrol and its derivatives may be considerably modified in an animal experiment.

Our study on various resveratrol derivatives revealed distinct mechanisms of their cytoprotective effect. None of the analogs completely shared the autophagy-dependent cytoprotection with a balanced effect on ROS level and mitochondria of resveratrol. Monomethyl resveratrol was the most similar to the parent compound, though autophagy induction played a less prominent role in its cytoprotective effect. Oxyresveratrol induced direct cytoprotection secondary to its antioxidant activity rather than activating mitophagy. The lack of free hydroxyl groups on trimethyl resveratrol annihilated its free radical scavenger activity, which was accompanied by autophagy induction, resulting in a weaker cytoprotective effect. Contrary to all other examined stilbenoids, dimethyl resveratrol was found to be cytotoxic to the fibroblasts. Understanding the structure–activity relationships and having a deeper insight into the mechanism of action of resveratrol and its analogs could bring us closer to develop them to cytoprotective drugs.

## 4. Materials and Methods

### 4.1. Reagents

Resveratrol, oxyresveratrol monomethylated and trimethylated resveratrol were purchased from TCI (Tokyo Chemical Industry, Tokyo, Japan). DMEM medium, stable glutamine and trypsin were obtained from Corning Inc. (New York, NY, USA), while fetal bovine serum (FBS) was purchased from BioSera (Nuaille, France). 7-Amino-4-methylcoumarin, fluorogenic caspase substrate (Ac-DEVD-AMC), 2′,7′-dichlorofluorescein diacetate (DCFDA), acridine orange (AO), phosphate inhibitor cocktail 2 and 3, Nonidet P40 and buffer constituents were purchased from Sigma-Aldrich (St. Louis, MO, USA). 5,5′,6,6′-tetrachloro-1,1′,3,3′-tetraethylbenzimidazolocarbocyanine iodide (JC-1), hydroethidine (HE) Mitotracker Green and Hoechst 33342 dye, 4–12% and 4–20% gradient PAGE-SDS gels, PVDF transfer membranes and ECL reagent were supplied by ThermoFisher Scientific (Waltham, MA, USA). Antibodies for Western blot were purchased from Cell Signaling Technology (Danvers, MA, USA).

Tested compounds were dissolved in dimethyl sulfoxide (DMSO) and used in the cell culture medium to provide 0.5% final DMSO concentration. Control cells were treated with the same concentration of DMSO.

Pregnant NMRI mice for cell culture establishment were supplied by Toxicoop, Gödöllő, Hungary. All animal procedures were in accordance with the EU Council Directives on laboratory animals (86/609/EEC).

### 4.2. Cell Culture and Treatment

Mouse embryonal fibroblast culture was established according to the CSH protocol [62].

MEF cells were maintained in DMEM supplemented with 10% FBS, 1% stable glutamine, 80 μg/mL gentamycin and 10 μg/mL ciprofloxacin and used between passages 3 and 5. Experiments were performed in 6 cm cell culture dishes and 24-well or 96-well cell culture plates. The cells were seeded in 40,000 cells/cm^2^ density and used after reaching confluency. Apoptosis was induced by the withdrawing of FBS for 3 h. Resveratrol, its analogs, and the autophagy inhibitors were added simultaneously with serum deprivation.

### 4.3. LDH Release Viability Assay

After a 6 h treatment period, aliquots of the cell culture medium were collected for determination of released LDH. The remaining cells were lysed by the addition of Triton X100 to a final concentration of 1% to estimate intracellular LDH activity. LDH activity was determined by CytoTox-ONE Homogeneous Membrane Integrity Assay (Promega, Madison, WI, USA) according to the manufacturer’s instructions, and fluorescence was measured by Varioskan LUX Microplate spectrofluorometer at 530/590 nm.

### 4.4. Caspase 3 Activation Assay

After a 3 h treatment period, the cells were harvested with 0.25% trypsin-EDTA and washed with PBS. The cells were suspended in hypotonic lysis buffer containing 0.6% Nonidet P40 at 4 °C for 10 min; then, they were centrifuged at 2500× *g*, 4 °C for one minute and the supernatant was collected [63].

Caspase 3 activation was determined by using its fluorogenic substrate (Ac-DEVD-AMC). Cytosol extract was incubated in the presence of substrate for 3 h followed by determination of the AMC formed by its fluorescence at excitation and emission wavelength of 360 and 460 nm, respectively, by using a Varioskan LUX Microplate spectrofluorometer (ThermoFisher Scientific). The amount of the liberated AMC was normalized to the total protein content of the sample measured by Bradford’s method [64].

### 4.5. Determination of ROS Generation and Mitochondrial Membrane Depolarization

After treatment, the cells were washed with PBS and harvested by trypsinization. After washing with PBS, the cells were incubated for 30 min in the presence of HE (1 μM in PBS) and DCFDA (2 μM in PBS) or JC-1 (5 μM in PBS) to detect ROS generation and mitochondrial membrane depolarization, respectively. Fluorescence intensity of the stained cells was determined at 485/530 and 530/590 nm in a Varioskan LUX Microplate spectrofluorometer. Depolarization of mitochondrial membrane potential was calculated as an increase in the green/red fluorescence ratio.

### 4.6. Mitochondrial Mass Measurement

The cells were seeded into a 96-well plate and treated with different concentrations of resveratrol analogs. The cells were incubated with MitotrackerGreen (200 nM) and Hoechst 33342 (2 nM) in HBSS for 30 min. The fluorescence intensity of the stained cells was determined from the bottom at 360/485 and 485/530 nm in a Varioskan LUX Microplate spectrofluorometer and the results were given as a ratio between green and blue fluorescence.

### 4.7. Acridine Orange Staining of the Acidic (Autophago-)Lysosomes

After treatment, the living cells were stained without fixation with acridine orange (AO, 1 μM in PBS) for 15 min in a 24-well plate. Basic molecule acridine orange accumulates in acidic vacuoles, where it exhibits red fluorescence, while nucleic acids in the nucleus and cytosol are stained green. After washing the excess dye with PBS, photos were taken by a multicolor digital camera using 485 nm excitation wavelength in an epifluorescent microscope equipped with 10× objective and 10× ocular. Images of all emitted wavelength were analyzed with FIJI [65], red and green channels were separated, and red fluorescence intensity/cell pixels was calculated (after using median autothreshold masking method).

### 4.8. Western Blot Analysis of Autophagy-Related Proteins

Cytosol extract samples (prepared as written in 4.3) were treated with Laemmli buffer at 95 °C for 5 min. An aliquot corresponding to 30 μg protein was subjected to SDS-PAGE separation on 4–20% or 4–12% gradient gels. After semi-dry blotting to PVDF, membrane pULK, ULK, LC3 and p62/SQSTM1 proteins were stained by primary antibodies in 500–1000× dilution and visualized by HRP conjugated anti-mouse and anti-rabbit secondary in 2000× dilution. Enhanced chemiluminescence signal was detected by a ChemiOnly imaging system (VWR International, Radnor, PA, USA).

### 4.9. Statistical Analysis

The data were analyzed by GraphPad Prism 8 (GraphPad Software, La Jolla, CA, USA). Concentration–response curves were constructed by the least square nonlinear regression method. One-way ANOVA followed by Dunnett’s post hoc test were used for data analysis. All data are presented as means ± SD and in the case of EC_50_ values, 95% confidence intervals were given of at least three parallel measurements. Differences were considered significant if *p* < 0.05.

## Data Availability

Data are contained within this article.
